# “I Can Read These Colors.” Orthographic Manipulations and the Development of the Color-Word Stroop

**DOI:** 10.3389/fpsyg.2012.00594

**Published:** 2013-01-08

**Authors:** Marie Arsalidou, Alba Agostino, Sarah Maxwell, Margot J. Taylor

**Affiliations:** ^1^Diagnostic Imaging, Hospital for Sick Children, University of TorontoToronto, ON, Canada; ^2^Neurosciences and Mental Health, Research Institute, Hospital for Sick Children, University of TorontoToronto, ON, Canada; ^3^Department of Psychology, Ryerson UniversityToronto, ON, Canada

**Keywords:** color-word Stroop, orthographic manipulation, children, interference, facilitation

## Abstract

The color-word Stroop is a popular measure in psychological assessments. Evidence suggests that Stroop performance relies heavily on reading, an ability that improves over childhood. One way to influence reading proficiency is by orthographic manipulations. To determine the degree of interference posed by orthographic manipulations with development, in addition to standard color-Words (purple) we manipulated letter-positions: First/last letter in correct place (prulpe) and Scrambled (ulrpep). We tested children 7–16 years (*n* = 128) and adults (*n* = 23). Analyses showed that Word- and First/last-incongruent were qualitatively similar, whereas Word-congruent was different than other conditions. Results suggest that for children and adults, performance was hindered the most for incongruent and incorrectly spelled words and was most facilitated when words were congruent with the ink color and correctly spelled. Implications on visual word recognition and reading are discussed.

## Introduction

The color-word Stroop task (Stroop, 1935) is a widely used measure that has been theorized to be an index of executive functioning such as interference control (e.g., van Mourik et al., 2005), selective attention and cognitive flexibility (e.g., Homack and Riccio, 2004; Charchat-Fichman and Oliveira, 2009), and response inhibition (Pocklington and Maybery, 2006). The Stroop task requires an individual to identify the ink color of stimuli as quickly as possible. Typically, participants are asked to name the color of the ink, of a list of “X”s (color-baseline condition) or the color of the ink of congruent color-words (i.e., the word red written in red ink; congruent condition). In the Stroop condition, the color of the ink is incongruent with the written word (i.e., the word blue written in red ink). Research consistently finds that it takes longer to name the color of the ink in the incongruent, Stroop condition. Many versions of the Stroop have been designed, such as the number Stroop and the emotional Stroop (MacLeod, 1991). When considering only the prototypical color-word Stroop, relative to the hundreds of adult studies, investigations over early development are scarce. Learning to read is a key contributor for detecting this effect, and as the color-word Stroop contains words it lends itself to orthographic manipulations. The main purpose of this study was to examine the effects of orthographic manipulation (i.e., changing letter-positions in color-words) on interference elicited by the Stroop task developmentally.

Comalli et al. (1962) were the first to use the Stroop with children and adults ranging from 7 to 80 years old (*N* = 235). Using 100-item cards they showed (a) colored rectangles (color-baseline), (b) color-words in black ink, and (c) color-words written in incongruent colors. Participants became progressively faster in responding to the three conditions as a function of age, but they were slowest on the incongruent colors. A large body of clinical and experimental research uses the color-word Stroop, such as in detecting deficits in inhibition in individuals with attention deficit disorder (e.g., Homack and Riccio, 2004; Schwartz and Verhaeghen, 2008 for meta-analyses). The majority of the studies using the color-word Stroop are individual difference rather than developmental studies. We found relatively few reports that examined three or more age groups of typically developing children and adolescence using the Stroop (Comalli et al., 1962; Schiller, 1966; Berninger et al., 1991; Armengol, 2002; Leon-Carrion et al., 2004; Pritchard and Neumann, 2004; Peru et al., 2006; Charchat-Fichman and Oliveira, 2009; Polderman et al., 2009), overall showing a negative relation between age and performance on the Stoop (i.e., as age increases, response times decrease).

Inhibitory control, assessed with measures other than the Stroop (e.g., Stop signal), also shows a protracted development from childhood to adulthood (Williams et al., 1999; Bedard et al., 2002; Davidson et al., 2006), although some suggest it develops very early (by grade 2; Schachar and Logan, 1990; Christ et al., 2001). It appears, however, that the rate at which inhibition develops changes as a function of age (Luna and Sweeney, 2004; Best et al., 2009; for reviews). Specifically, improvements in inhibitory abilities are easily detected in pre-school children (Montgomery and Koeltzow, 2010) yet improvements are also reported for middle-school children and adolescents (Leon-Carrion et al., 2004; Luna and Sweeney, 2004), with 13-year-olds still not attaining complete adult levels (Davidson et al., 2006). On average, younger children (6–8 years) are about 50 ms slower in stopping a prepotent response than older children (9–12) who in turn are about 30 ms slower than adolescents (13–17 years; Williams et al., 1999). The latter results are consistent with neuroimaging findings showing that the pre-frontal cortex, an area highly correlated with executive functions, continues to develop through childhood and adolescence (Kolb and Whishaw, 2003) and this protracted maturation is reflected in the development of inhibitory abilities (Luna, 2009). Meta-analysis evidence verifies that the pre-frontal cortex plays a key role on the Stroop performance in adults (Laird et al., 2005).

Developmental functional magnetic resonance imaging (fMRI) studies using the Stroop (Adleman et al., 2002; Marsh et al., 2006) have used a sub-vocal response modality, which the authors acknowledged was a limitation in their study due to lack of task compliance assessment during scanning (Adleman et al., 2002). Sub-vocal responding may also increase voluntary or involuntary movements that could compromise the quality of brain images. Therefore, with a future aim to study the brain correlates of orthographic effects in the Stroop, we designed our protocol by modifying the Stroop paradigm to be compatible for use with fMRI and incorporated a speeded, manual response.

Apart from presentation and response modality modifications, the Stroop paradigm has been adapted widely to investigate the effect of interference in many domains and in different contexts (MacLeod, 1991, 2005 for comprehensive reviews). Past studies modified the Stroop by altering pronounceability of non-words (e.g., “hrwd” and “swal”) and the meaning of words in relation to their color (e.g., “carrot” and “chair”); these were found to affect the intensity of interference (e.g., longer responses to “carrot” when written in incongruent ink color; MacLeod, 1991). Also, using only certain letters of the color word (e.g., the first letter; Regan, 1978 or the first three letters; McCown and Arnoult, 1981) were enough to elicit interference in adults; comparable investigations were not completed with children. Relevant developmental work was performed by Berninger et al. (1991), who showed children in grades 2, 4, and 6, color-words in which either two letters of the word (e.g., gre*en*, “en” printed in red) or single-letter combinations (e.g., g*r*een, “r” printed in red) were printed in incongruent colors, as well as whole words (e.g., green printed in red). The authors observed that students’ responses were slowest in the following order: word > single-letter > two-letter cluster. Berninger et al. (1991) did not include stimuli with transposed letters (i.e., students viewed the whole-word spelled correctly). We are not aware of any studies that directly manipulated orthography of the color-words in the Stroop to examine age-related effects.

The ability to read is clearly a component for observing the Stroop effect, as children under the age of six do not experience this effect (e.g., Comalli et al., 1962; Peru et al., 2006), but at the age of seven this effect is observed (e.g., Comalli et al., 1962; Armengol, 2002; Peru et al., 2006). Learning to read is a critical achievement for children, which requires concurrent coordination of semantic, phonological, and orthographic features (Ehri, 2005). According to phase theory (e.g., Ehri, 1995, 2005) all words, via appropriate practice, are read through sight. Sight word reading, as it is referred to, undergoes four successive phases: pre-alphabetic, partial, full, and consolidated alphabetic phases (Ehri, 1995, 2005). Using various measures of reading development (e.g., test of alphabetical knowledge, vocabulary, and reading comprehension), Vellutino et al. (2007) proposed a comprehensive model of reading proficiency in younger (grades 2–3) and older readers (grades 6–7), showing the multifaceted aspects of reading. Across development, reading becomes increasing automatic in grades 1–5 (Paris, 2005), with practiced words attaining mastery sooner than others (Ehri, 2005). Reading skills follow a sigmoid (S-) growth function; learning begins slowly, followed first by a sharp learning curve and then by slow improvements toward a plateau (Paris, 2005). Specifically, children read about 50 words correctly per minute when they start to read (e.g., grade 1; 5–6 years) and improve by about 13 more words per minute, per year, up to grade 5 (10–11 years; Paris, 2005). Overall, reading is a complex ability that is typically achieved, via practice, in the first decade of life.

Intricate processes that underlie reading ultimately become automatic. Adult research clearly shows that letter position in a word has an effect on its readability (Grainger and Van Heuven, 2003). Grainger and Whitney (2004) wrote “Does the huamn mnid raed wrods as a wlohe?”; by summarizing research on this topic they explained that printed words are encoded in a special way, making reference to studies examining two phenomena: (a) relative-position priming and (b) transposition priming. Primes that either retain their position pattern (e.g., “mthr” prime for “mother”) or have adjacent letters transposed (e.g., “mohter” prime for “mother”) lead to the targets being processed faster. Although, letter position has been manipulated to study its effect on inhibition in adults using the Stroop (Regan, 1978; McCown and Arnoult, 1981), there are no reports of such effects in children and adolescence.

Here we investigated interference based on orthographic manipulations in the Stroop across development. Specifically, we examined (a) orthographic effects on interference elicited by the Stroop and (b) age effects on performance as they relate to the different orthographic manipulations. As letter position affects readability of a word, we anticipated that it would, in turn, affect interference experienced in the color-word Stroop, in children and adults. We included whole color-words, words that retained the position of the first and last letters and scrambled color-words in congruent and incongruent trials. We expected that words that retained the position of the first and last letters would elicit more interference than the scrambled words. In addition, we wanted to validate the parameters of our protocol (e.g., stimulus presentation intervals and manual response) to confirm that we could successfully detect the interference effects and in turn establish its suitability for neuroimaging methods.

## Materials and Methods

### Participants

We present data from 151 participants. Children were recruited from Toronto public schools, enrolled in mainstream classes, from grades 2 (7–8 years), 4 (9–10 years), 6 (11–12 years), 8 (13–14 years), and 10 (15–16 years), and adults (*n* = 23, ages 19–30 years) were recruited from the community (Table 1). None of the participants had any history of neurological or psychiatric disorders. All school-aged participants were recruited from the classrooms and their teachers confirmed verbally that none of those included in this study had reading difficulties, dyslexia, or learning disabilities. All participants provided informed consent; for the children, this included consent from the child’s parent. The Research Ethics Board at the Hospital for Sick Children approved all procedures.

**Table 1:** **Participant characteristics and performance**.

		Grade 2		Grade 4		Grade 6		Grade 8		Grade 10		Adults		Full total	
	*N* (female)	19 (15)		26 (17)		26 (12)		24 (17)		33 (22)		23 (14)		151 (97)	
	Age range	7.4–8.3		9.4–10.3		11.4–12.3		13.3–14.3		14.9–16.2		20.21–29.3		7.4–29.3	
	Age (*M* ± SD)	7.86 ± 0.25		9.76 ± 0.29		11.79 ± 0.25		13.7 ± 0.32		15.5 ± 0.31		23.30 ± 2.6		13.87 ± 4.83	

Condition		*M*	SD	*M*	SD	*M*	SD	*M*	SD	*M*	SD	*M*	SD	*M*	SD
Color-baseline	RT	905	93	885	83	804	87	776	111	676	85	692	64	775	119
	Err	0.16	0.10	0.09	0.09	0.06	0.07	0.05	0.05	0.05	0.06	0.02	0.02	0.06	0.08
Word-congruent	RT	901	109	831	75	764	93	736	118	639	88	649	65	743	129
	Err	0.23	0.18	0.1	0.1	0.06	0.08	0.03	0.04	0.03	0.05	0.02	0.05	0.07	0.11
Word-incongruent	RT	962	106	912	73	886	92	863	111	742	87	736	77	842	123
	Err	0.33	0.23	0.18	0.16	0.12	0.13	0.16	0.15	0.08	0.1	0.02	0.03	0.14	0.15
First/last-congruent	RT	923	99	884	92	839	87	803	117	683	113	692	84	796	134
	Err	0.22	0.2	0.13	0.13	0.03	0.05	0.06	0.07	0.04	0.04	0.03	0.04	0.07	0.12
First/last-incongruent	RT	964	61	903	95	860	83	831	101	740	105	730	74	830	118
	Err	0.28	0.22	0.14	0.14	0.08	0.08	0.08	0.11	0.04	0.07	0.02	0.05	0.10	0.14
Scrambled-congruent	RT	953	99	876	74	856	102	789	120	683	106	684	70	797	137
	Err	0.3	0.22	0.13	0.11	0.06	0.11	0.05	0.06	0.05	0.05	0.02	0.3	0.09	0.13
Scrambled-incongruent	RT	910	103	898	85	823	106	780	124	693	109	709	74	795	131
	Err	0.34	0.23	0.1	0.11	0.05	0.09	0.05	0.07	0.04	0.05	0.02	0.03	0.09	0.14
Word difference*	RT	57	116	59	117	82	68	87	90	66	66	45	73	67	88
First/last difference	RT	60	91	50	85	56	80	55	77	63	80	40	54	54	77
Scrambled difference	RT	6	135	45	59	19	93	4	71	16	100	21	68	19	89
Word facilitation	RT	−4	94	−21	63	−40	70	−40	69	−37	58	−43	52	−32	67

RT, response times in milliseconds; Err, proportion of errors. Difference scores were calculated by subtracting color-baseline from the incongruent scores. *Word difference, corresponds to what is typically referred to as interference score. Word facilitation was calculated by subtracting color-baseline from Word-congruent. A MANOVA was used to assess differences in error rates among the six age groups. This analysis yielded a significant main effect for age group [Wilk’s **Λ** = 0.53, *F*_(35, 725)_ = 3.68, *p* < 0.000]. Grade 2 made more errors when compared to the older groups, thus, we examined whether latencies differed for correct and incorrect responses for grade 2. In a 2 response (correct and incorrect) by seven condition MANOVA we found no significant difference in RT for correct and incorrect responses.

### Materials and method

Four colors were chosen for this task. Criteria for color selection were based on the color-word length and how commonplace the color was. Orange, yellow, purple, and white were selected as they contained five or more letters, which allowed flexibility in manipulating the orthography and generating the stimuli. Also, we carefully selected the hues such that the colors were easily recognizable and distinguishable by the participants. Participants were first asked to read four color-words (orange, yellow, purple, and white) printed in black ink to verify proficiency in reading these words and to name the color of rectangular blocks to verify proficiency in identifying the colors. All participants were able to accurately read and name colors.

We used a computerized, speeded manual response protocol. To familiarize participants with the timing of the task and location of the four color buttons on the keyboard they completed a 16-trial training session. Training stimuli were presented for 1500 ms with an inter-stimulus interval of 500 ms. Participants responded successfully to training: 97% made two or fewer errors.

**Figure 1:**
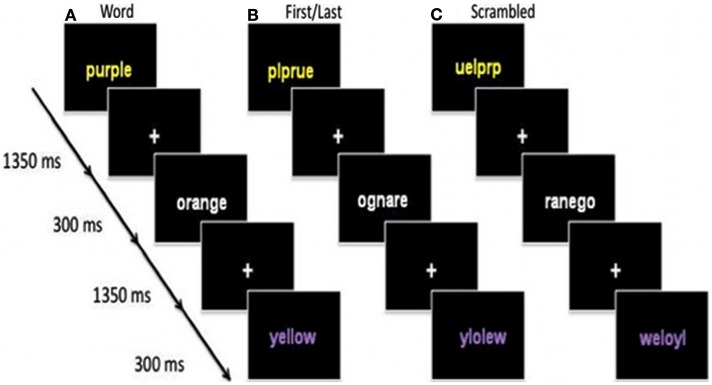
**Examples of incongruent stimuli for the three word-types. (A)** Incongruent colour words, **(B)** Incongruent scrambled colour words with the first and last letter in place and **(C)** Incongruent scrambled colour words.

We used three word-type manipulations: (a) Word, (b) First/last letter in place, and (c) Scrambled (Figure 1). Task conditions consisted of color-words written in either congruent (e.g., yellow written in yellow ink) or incongruent (yellow written in purple ink) color. In the First/last condition, the first and last letters of the color word were kept in place while the middle letters were scrambled and the words were either congruent (e.g., ylloew written in yellow ink) or incongruent (e.g., yleolw written in purple ink) with ink color. The Scrambled condition consisted of scrambled-congruent (e.g., wlyloe written in yellow ink) and incongruent (e.g., wylleo written in purple ink) color-word pairings, which was added to account for the visual presentation of letters arranged in a non-word format. The Color-baseline condition consisted of a line “x”s printed in the same four colors. Stimuli were presented on a gray background. Care was taken to ensure each color appeared with equal frequency across the conditions and that stimuli would not positively or negatively prime the subsequent stimulus, which was a key reason for using a four alternative force choice key press task. Stimuli were presented for 1350 ms with an inter-stimulus interval of 300 ms.

Each of the six conditions, plus Color-baseline, consisted of two blocks of 10 trials pseudo-randomly presented resulting in a total of 140 trials. Participants were instructed to respond to ink color of stimuli as quickly as possible while maintaining accuracy by pressing colored keys on a standard keyboard; we used colored stickers on the relevant keys to remove demands on memory. Using Presentation software (Neurobehavioral Systems), we recorded both accuracy and RTs.

### Data screening and analyses

Prior to analyses, scores were examined through SPSS programs for accuracy of data entry, missing values, and the assumptions of univariate and multivariate analyses. Pairwise linearity was checked using scatterplots and found to be satisfactory.

Trials were coded as incorrect if the participant failed to respond or provided an incorrect response. The dependent variable was the average RT per item (in milliseconds). Individual RT trials were based on trimmed raw data (i.e., excluded if RT was less than 200 ms or greater than 3 SD from the mean). Eight participants [six in grade 2 (7–8 years, 4 females) and two in grade 4 (9–10 years, 2 males)] were found to be outliers and were not included in our sample or in analyses, as they performed at chance level (i.e., below 60% correct). Statistical tests were performed on data from 151 participants. Age effects were tested using multivariate analyses of variance, in which age was treated as a categorical variable. To test the orthographic effects of interference among conditions we conducted planned contrasts with Bonferroni multiple comparison control. Structural equation modeling (SEM) and correlational methods were conducted to examine the relation of age with interference in each condition; these analyses treated age as a continuous variable.

## Results

### Age effects

**Figure 2:**
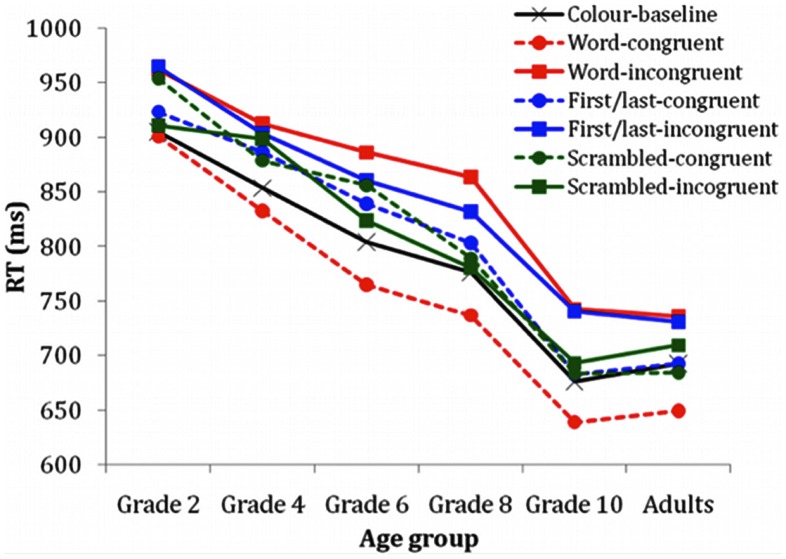
**Response times as a function of age and word-type**.

A MANOVA assessed RTs across age groups on a linear combination of performance in Color-baseline and incongruent and congruent trials for all three conditions (i.e., Word, First/Last, and Scrambled; Figure 2; Table 1). By forming linear combinations of dependent variables, this test identifies differences among the age groups. A significant effect was found, Wilk’s **Λ** = 0.35, *F*_(35, 587)_ = 4.78, *p* < 0.0001, multivariate η^2^ = 0.19. Table 2 summarizes significant *post hoc* age group differences. Specifically, Color-baseline, Word-congruent trials and Scrambled-congruent trials showed the same developmental patterns. Most differences in RT were observed earlier in development; performance of grade 10 children did not differ from that of adults.

**Table 2:** **Significant *Post hoc* Age differences per trial type**.

	Color-baseline						First/last-congruent						Word-incongruent						First/last-incongruent						Scrambled-incongruent					
	Word-congruent					
	Scrambled-congruent					
Grades	2	4	6	8	10	A	2	4	6	8	10	A	2	4	6	8	10	A	2	4	6	8	10	A	2	4	6	8	10	A
4	*	–					.	–					.	–					.	–					.	–				
6	*	.	–				.	.	–				.	.	–				*	.	–				.	.	–			
8	*	*	*	–			*	*	.	–			*	.	.	–			*	*	.	–			*	*	.	–		
10	*	*	*	.	–		*	*	*	*	–		*	*	*	*	–		*	*	*	*	–		*	*	*	*	–	
A	*	*	*	.	.	–	*	*	*	*	.	–	*	*	*	*	.	–	*	*	*	*	.	–	*	*	*	.	.	–

*Significant at *p* = 0.05; .no significant difference; A, adults.

### Word-type differences among *incongruent* trials

To determine RT differences among word-type conditions we performed a series of contrasts, collapsed across groups (Table 1). Word-incongruent RTs and First/last-incongruent RTs were marginally different (*t* = 1.94, DF = 150, *p* = 0.054, partial η^2^ = 0.03). Word-incongruent RTs and Scrambled-incongruent RTs yielded a significant difference (*t* = 6.63, DF = 150, *p* < 0.0001, partial η^2^ = 0.24). This contrast yielded a large effect size, as did the contrast between First/last-incongruent RTs and Scrambled-incongruent RTs (*t* = 5.72, DF = 150, *p* < 0.0001, partial η^2^ = 0.18). These results suggest that on average participants required significantly more time to complete incongruent Word and First/Last than Scrambled trials.

### Word-type differences among *congruent* trials

A series of comparisons were conducted among the three sets of congruent trials. Unlike the incongruent trials, the comparison between Scrambled and First/last-congruent was not significantly different (*t* = 0.33, DF = 150, *p* = 0.74; Figure 2). Participants were significantly faster on the Word-congruent than First/last-congruent (*t* = 7.73, DF = 150, *p* < 0.000, partial η^2^ = 0.27) and Scrambled-congruent (*t* = 8.75, DF = 150, *p* < 0.000, partial η^2^ = 0.34).

### Relations among RT scores and age

We examined the relations between the various scores and age (Table 3). All scores remained significant even after controlling for the effects of age and RT to Color-baseline trials. Together these findings suggest that age and Color-baseline RT (i.e., responding to a stimulus that only included x’s) do not fully account for the relations among the scores on the congruent and incongruent trials.

**Table 3:** **Correlations among scores and age**.

	1	2	3	4	5	6	7	8
1. Age		–	–	–	–	–	–	–
	–	–	−0.24**	−0.32**	−0.24**	−0.32**	−0.30**	−0.20*
2. Color-baseline			0.78**	0.60**	0.71**	0.68**	0.75**	0.64**
	−0.56**	–	–	–	–	–	–	–
3. Word-congruent				0.60**	0.71**	0.64**	0.76**	0.65**
	−0.59**	0.86**	–	0.32**	0.38**	0.29**	0.45**	0.35**
4. Word-incongruent					0.64**	0.70**	0.68**	0.65**
	−0.59**	0.73**	0.74**	–	0.42**	0.56**	0.50**	0.47**
5. First/last-congruent						0.70**	0.77**	0.67**
	−0.57**	0.81**	0.81**	0.76**	–	0.45**	0.54**	0.42**
6. First/last-incongruent							0.71**	0.73**
	−0.60**	0.79**	0.77**	0.81**	0.80**	–	0.47**	0.55**
7. Scrambled-congruent								0.72**
	−0.61**	0.84**	0.84**	0.80**	0.85**	0.82**	–	0.50**
8. Scrambled-incongruent								
	−0.53**	0.75**	0.76**	0.76**	0.77**	0.82**	0.81**	–

Correlations above the diagonal controlling for age (top value) and color-baseline (bottom value), zero-order correlations below the diagonal. *N* = 151, two-tailed, **p = 0.001, *p = 0.01.

**Figure 3:**
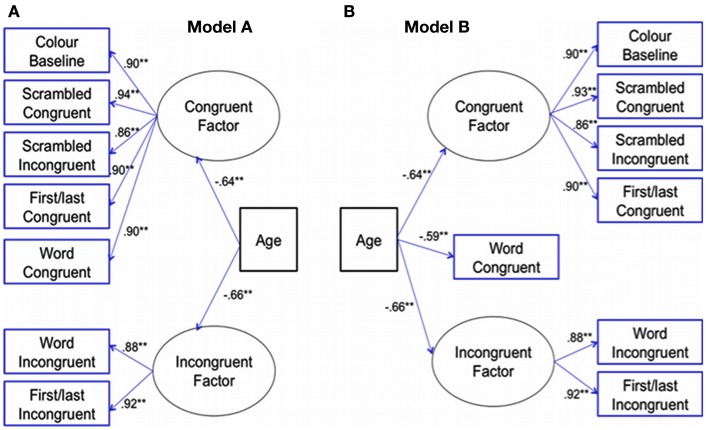
**Path models depicting latent factors predicted by age**. Note **(A)** Depicts Word-incongruent and First/last-incongruent loading onto a latent Incongruent factor, whereas the rest conditions load significantly onto a latent congruent factor. **(B)** Depicts a path with a better fit showing the Word-congruent significantly loading on its own; rest were same as Model A.

Thus, a path model was used to determine qualitative differences in performance among word-types (Figure 3A). We hypothesized that shared variance between Word-incongruent and First/last-incongruent would load onto an incongruent factor. Scrambled-incongruent, the three congruent sets of trials and Color-baseline were hypothesized to load significantly onto a congruent factor. Age was a directly linked to both factors and their error terms were correlated. Using maximum likelihood estimate this model yielded a good fit to the data, as shown by a non-significant chi-square value, χ^2^ (18, *N* = 151) = 28.30, *p* = 0.059, root mean squared error of approximation (RMSEA) = 0.06, comparative fit index (CFI) = 0.99, and normed fit index (NFI) = 0.98. Standardized factor loadings for the indicator variables are presented in Figure 3A and were significant at *p* < 0.001.

An alternative model B was also tested to assess whether Word-congruent was better positioned as a factor on its own as all age groups produced faster RTs on Word-congruent, suggesting that the condition might be qualitatively different (i.e., facilitating; congruent word would speed up the identification of the ink color). Model B depicted in Figure 3B posited a three factor model. This alternative model also yielded a very good fit to the data as the chi-square was non-significant, χ^2^ (16, *N* = 151) = 22.30, *p* = 0.14; RMSEA = 0.05; CFI = 0.99, and NFI = 0.98. Positioning Word-congruent as a facilitating construct appeared to improve the fit of the model. Therefore, a chi-square difference test was conducted, comparing model A with model B. The chi-square for this model was equal to 28.30 − 22.3 = 6.00 which, with a 2 DF was significant (*p* = 0.05). Interestingly, age was significantly linked to all three constructs; however, age accounted for the least amount of variance in the Word-congruent condition.

## Discussion

This study determined the extent to which orthographic manipulations influence interference control across development. We manipulated color-word orthography in a Stroop task and examined performances in ages 7–30 years. There were three main findings:

(a) Age was a significant predictor for all factors, incongruent, congruent, and facilitating. A novel age-related finding was that unlike younger age groups, late adolescent’s behavioral performance was adult-like, cautioning against averaging over age ranges including children and adolescents.
(b) Performances on Word-incongruent and First/last-incongruent trials were qualitatively similar, suggesting that children, like adults, attempt to read pseudo-color-words with the first and last letter in place. This suggests that children detected the wrong spelling in color-words and their performance was delayed as they strived to recover from the incongruent ink color, similar to what they experienced with correctly spelled color-words.
(c) Performance on Word-congruent was different from performances on First/last-congruent, Scrambled-congruent, Scrambled-incongruent, and Color-baseline, which were all qualitatively similar. This is in agreement of the hypothesis that Word-congruent is facilitating, which we showed to be facilitating for children as well.

### Age effects

We examined the effects of age on task performance in children and young adults. Children in grade 2 (7–8 years-olds), the youngest age group, were significantly slower than grade 8s (13–14 years) and older for Word-incongruent and grade 6s (11–12 years) and older for First/last-incongruent; suggesting a sharper decrease in response time for First/last-incongruent as a function of age (Table 2). RT differences were not observed for children in grades 4 (9–10 years), 6, and 8 for Word-incongruent; however, children in grades 4 and 8 differed for First/last-incongruent. Results of age group differences on the congruent trials revealed that Word-congruent and Scrambled-congruent forms echoed the developmental pattern found in the Color-baseline. We highlight that adults and students in grade 10 (15–16 years) exhibited comparable response times. In the developmental literature reviewed, only one study reported normative data for late adolescence (ages 15–17; Leon-Carrion et al., 2004). Despite the lack of normative data, particularly during the adolescent years, some clinical studies average over large age ranges (e.g., Reeve and Schandler, 2001; White et al., 2001; Favre et al., 2009; Peterson et al., 2009). We showed that adolescents’ performance was adult-like; thus, we recommend against averaging over large age groups of children, particularly when younger children are included in the same age group as late adolescents (e.g., 15–16 years).

In the path analyses, age was positioned as a predictor for all constructs and these were found to be significant (Figure 3). Specifically, age accounted for slightly more variance in the Incongruent factor and for the least amount for variance for the Word-congruent. Previous research on the Stroop documented that children became progressively faster as they responded verbally to stimuli (Comalli et al., 1962; Schiller, 1966; Berninger et al., 1991; Armengol, 2002; Leon-Carrion et al., 2004; Pritchard and Neumann, 2004; Peru et al., 2006; Charchat-Fichman and Oliveira, 2009; Polderman et al., 2009), and this was what we also found with speeded manual responses (Figure 2; Table 3). Adult studies suggest that greater interference is sometimes observed with vocal compared to manual responses (White, 1969; Redding and Gerjets, 1977; MacLeod, 1991). Although the response times we observed were much faster (i.e., under 1 s, Figure 2) than those requiring verbal response (Comalli et al., 1962; Schiller, 1966; Berninger et al., 1991; Armengol, 2002; Leon-Carrion et al., 2004; Pritchard and Neumann, 2004; Peru et al., 2006; Charchat-Fichman and Oliveira, 2009; Polderman et al., 2009), relations with age were strong; we showed that age shared approximately 33% of the variance with all conditions (Table 3). Inter-correlations with conditions were stronger, showing greater common variance ranging from 53 to 74%. We also accounted for the variance of age; however correlations remained significant among conditions, albeit the strength of the relations decreased (Table 3, upper diagonal-top value). This suggests that age alone cannot account for the variance shared among conditions. As response times improve with age regardless of task, then response time to Color-Baseline condition could account for these relations. When the correlations controlled for responses to Color-baseline (i.e., controlled for the ubiquitous age-related decreases in RTs), the strength of the relations decreased, but the outcome remained significant (Table 3, upper diagonal-bottom value). Significant partial correlations may be attributed to individual differences and related executive processing or working memory. Working memory, the ability to hold and manipulate information for a short time, improves with age. Particularly, research shows that working memory capacity is better assessed by measures that contain task-irrelevant features (Arsalidou et al., 2010), thus likely contributes to the performance changes we observed.

Overall, it appears that responses to the Stroop task, linked as it is to executive functions such as inhibition, continue to develop throughout middle-childhood and adolescence (Comalli et al., 1962; Williams et al., 1999; Bedard et al., 2002; Luna and Sweeney, 2004; Peru et al., 2006; Best et al., 2009). Although the traditional response modality in the Stroop is vocal, this poses limitations when applied with imaging technologies that are susceptible to movement artifacts. Sub-vocal responses used previously in developmental fMRI studies with children preclude assessment of task compliance or performance during scanning (Adleman et al., 2002; Marsh et al., 2006). Our data show that speeded manual responses accurately capture performance trajectories in children.

### Effects of orthographic manipulations

To assess the effects of orthography, we used three word-type conditions: whole color-words, color-words with first and last letters in place and scrambled color-words; all had both congruent (ink color consistent) and incongruent (ink color inconsistent) trials. For incongruent trials, RTs were affected by word-type, such that Word > First/last > Scrambled (Figure 2; Table 1); the largest effect size was observed when Word was compared to Scrambled, suggesting that the Scrambled-incongruent was the most different of the incongruent trials. For congruent trials, response times on the Scrambled and First/last-congruent were not significantly different; however these trials differed significantly from Word-congruent, with moderate effect sizes. In agreement with previous results (MacLeod, 1991), this suggests that Word-congruent trials may be facilitating. Children, as adults, experienced the least interference for Word-congruent. The highest interference was experienced during Word-incongruent, although First/last-incongruent had very similar performance curves.

Path analyses showed that Word and First/last-incongruent trials were qualitatively different from the rest of the trials, and loaded onto the same Incongruent factor (Figure 3). This suggests that our participants, all experienced interference when the first and last letters retained the correct position in the color-word. As Stroop interference is produced by the conflict between the tendency to read the color-word and naming the ink color, these data suggest that children as young as seven were “reading” the pseudo-color-words with the first and last letter in the correct place. This may also suggest that children recognized that these words were spelled wrong, and in turn experienced similar incongruence effects observed with correctly spelled color-words; the Scrambled-incongruent condition did not elicit this effect. Research, primarily based on adults, showed that letter position has an effect on the readability of words (Grainger and Van Heuven, 2003; Grainger and Whitney, 2004). Adult Stroop studies demonstrated that retaining the first letter interferes more than retaining the middle or last two letters of color-words (Singer et al., 1975). A similar finding was observed by Regan (1978) who showed that the first letter of color word could cause interference. Even if the first letter of a non-color-word matches the color-word, interference is generated in adults (e.g., Marmurek et al., 2006). Although we have not come across a study that examined this effect in children, developmental studies that manipulated letter-position in reading tasks, emphasize primarily its relation to lexical stress in the process of learning (Bowman and Treiman, 2002; Perea and Estevez, 2008; Ktori and Pitchford, 2009; Arciuli et al., 2010). These findings were linked to the work of Ehri (1995) on the phases of reading development, which suggests that ultimately all words become automatic and are read through sight. In the case of the current experiment, if the children were familiar with the color-words and were not trying to read them, we would not observe interference either with the whole word or the words with first/last letter in place. Our data suggest that at 7-years of age (grade 2) children were attempting to read using similar whole-word cues, and experienced Stroop incongruence effects as older children and adults, giving support to the sight word reading hypothesis (Ehri, 1995, 2005).

The path analyses also showed that Word-congruent trials were qualitatively different from all the other trials. The model that accounted for Word-congruent as a separate entity (Figure 3B) had a better fit to the data than the one that allowed for Word-congruent to load onto the Congruent factor (Figure 3A). This is consistent with the notion that response times are facilitated when the distractor color-word is the same as the ink color (MacLeod, 1991, 2005 for review). Usually, Stroop facilitation scores are calculated by subtracting RTs to Color-baseline from congruent conditions (Regan, 1978). Adult studies occasionally report Stroop facilitation scores to represent this effect (e.g., Stirling, 1979); however, these data are scarce developmentally. In a study with a small sample size – 9–13 years old children (*n* = 11) – a significant difference in Stroop facilitation was observed compared to adults, but not interference (Wright and Wanley, 2003). In a larger sample (11 year olds, *n* = 80; adults, *n* = 70) an effect of facilitation (comparing congruent vs. neutral condition) was only observed in children, not adults (Fagot et al., 2009). The only large developmental study that mentioned Stroop facilitation was by Charchat-Fichman and Oliveira (2009); however, they did not report facilitation scores in their sample. For completeness we report difference scores on facilitation (Table 1). The youngest children experience the least facilitation and these scores appear more adult-like by about grade 6 (Table 1).

Our findings are consistent with research that shows that children do not rely merely on rote memorization, but also rely on letter positions in reading (Bowman and Treiman, 2002; Peressotti et al., 2010). Adopting a multiple orthographic-phonological approach of teaching children to read had been found to facilitate learning, particularly in the early years (Hart et al., 1997). Brain research shows that visual word recognition elicits activity in the left fusiform gyrus, which is particularly affected by orthographic structure (Binder et al., 2006), and assimilates features during recognition of visual stimuli (Allison et al., 1994; Starrfelt and Gerlach, 2007; Arsalidou and Taylor, 2011). Thus, as children become expert readers, the fusiform gyrus may become more efficient or specialized. Even a year or two of practicing reading elicits a predisposition to reading words as a whole, as the First/last effect was present in the youngest children tested.

## Conclusion

Our primary finding indicates that children as young as seven can experience interference from words that only retain the position of first and last letters in color-words. This suggests that children process color-words as a whole, as is evident from the rate with which they can control irrelevant cues as they mature. Although performance trajectories were similar, and predicted by age, the underlying mechanisms for processing incongruent and congruent materials were qualitatively different. Characterizing congruency between color-word and ink color as facilitating generated a stronger model for predicting performance on this task and its relation with age. Our findings contribute to the understanding of the developmental relation among inhibition, interference control, orthography, and reading. The speeded, manual responses required in our protocol make it appropriate for use with neuroimaging technologies. Future work examining the brain correlates of orthographic manipulations will elucidate the brain mechanisms that underlie these relations over childhood.

## Conflict of Interest Statement

The authors declare that the research was conducted in the absence of any commercial or financial relationships that could be construed as a potential conflict of interest.

## References

[B1] Adleman N. E. Menon V. Blasey C. M. White C. D. Warsofsky I. S. Glover G. H. (2002). A developmental fMRI study of the Stroop color-word task. Neuroimage 16, 61–75 10.1006/nimg.2001.1046 11969318

[B2] Allison T. McCarthy G. Nobre A. Puce A. Belger A. (1994). Human extrastriate visual cortex and the perception of faces, words, numbers, and colors. Cereb. Cortex 4, 544–554 10.1093/cercor/4.5.544 7833655

[B3] Arciuli J. Monaghan P. Seva N. (2010). Learning to assign lexical stress during reading aloud: corpus, behavioral, and computational investigations. J. Mem. Lang. 63, 180–196 10.1016/j.jml.2010.03.005

[B4] Armengol C. G. (2002). Stroop test in Spanish: children’s norms. Clin. Neuropsychol. 16, 67–80 10.1076/clin.16.1.67.8337 11992229

[B5] Arsalidou M. Pascual-Leone J. Johnson J. (2010). Misleading cues improve developmental assessment of working memory capacity: the color matching tasks. Cogn. Dev. 25, 262–277 10.1016/j.cogdev.2010.07.001

[B6] Arsalidou M. Taylor M. J. (2011). Is 2+2=4? Meta-analyses of brain areas needed for numbers and calculations. Neuroimage 54, 2382–2393 10.1016/j.neuroimage.2010.10.009 20946958

[B7] Bedard A.-C. Nichols S. Barbosa J. A. Schachat R. Logan G. D. Tannock R. (2002). The development of selective inhibitory control across the life span. Dev. Neuropsychol. 21, 93–111 10.1207/S15326942DN2101_5 12058837

[B8] Berninger V. W. Yates C. Lester K. (1991). Multiple orthographic codes in reading and writing acquisition. Read. Writ. 3, 115–149 10.1007/BF00420030

[B9] Best J. R. Miller P. H. Jones L. L. (2009). Executive functions after age 5: changes and correlates. Dev. Rev. 29, 180–200 10.1016/j.dr.2009.05.002 20161467 PMC2792574

[B10] Binder J. R. Medler D. A. Westbury C. F. Liebenthal E. Buchanan L. (2006). Tuning of the human left fusiform gyrus to sublexical orthographic structure. Neuroimage 33, 739–748 10.1016/j.neuroimage.2006.06.053 16956773 PMC1634933

[B11] Bowman M. Treiman R. (2002). Relating print and speech: the effects of letter names and word position on reading and spelling performance. J. Exp. Child. Psychol. 82, 305–340 10.1016/S0022-0965(02)00101-7 12225758

[B12] Charchat-Fichman H. Oliveira R. M. (2009). Performance of 119 Brazilian children on Stroop paradigm-Victoria version. Arq. Neuropsiquiatr. 67, 445–449 10.1590/S0004-282X2009000300014 19623442

[B13] Christ S. E. White D. A. Mandernach T. Keys B. A. (2001). Inhibitory control across the life span. Dev. Neuropsychol. 20, 653–669 10.1207/S15326942DN2003_7 12002099

[B14] Comalli P. E. Jr. Wapner S. Werner H. (1962). Interference effects of Stroop color-word test in childhood, adulthood, and aging. J. Genet. Psychol. 100, 47–53 10.1080/00221325.1962.10533572 13880724

[B15] Davidson M. C. Amso D. Anderson L. C. Diamond A. (2006). Development of cognitive control and executive functions from 4 to 13 years: evidence from manipulations of memory, inhibition and task switching. Neuropsychologia 44, 2037–2078 10.1016/j.neuropsychologia.2006.02.006 16580701 PMC1513793

[B16] Ehri L. C. (1995). Phases of development in learning to read words by sight. J. Res. Read. 18, 116–125 10.1111/j.1467-9817.1995.tb00077.x

[B17] Ehri L. C. (2005). Learning to read words: theory, findings and issues. Sci. Stud. Read. 9, 167–188 10.1207/s1532799xssr0902_4

[B18] Fagot D. Dirk J. Ghisletta P. de Ribaupierre A. (2009). Adults’ versus children’s performance on the Stroop task: insights from ex-Gaussian analysis. Swiss J. Psychol. 68, 17–24 10.1024/1421-0185.68.1.17

[B19] Favre T. Hughes C. Emslie G. Stavinoha P. Kennard B. Carmody T. (2009). Executive functioning in children and adolescents with major depressive disorder. Child Neuropsychol. 15, 85–98 10.1080/09297040802577311 19089695 PMC2822399

[B20] Grainger J. Van Heuven W. J. B. (2003). “Modeling letter position coding in printed word perception,” in Mental Lexicon: “Some Words to Talk about Words”, ed. Bonin P. (New York: Nova Science Publishers).

[B21] Grainger J. Whitney C. (2004). Does the huamn mnid raed wrods as a wlohe? Trends Cogn. Sci. 8, 58–59 10.1016/j.tics.2003.11.006 15588808

[B22] Hart T. M. Berninger V. M. Abbott R. D. (1997). Comparison of teaching single or multiple orthographic-phonological connections for word recognition and spelling: implications for instructional consultation. School Psychol. Rev. 26, 279–297

[B23] Homack S. Riccio C. A. (2004). A meta-analysis of the sensitivity and specificity of the Stroop color and word test with children. Arch. Clin. Neuropsychol. 19, 725–743 10.1016/j.acn.2003.09.003 15288327

[B24] Kolb B. Whishaw I.Q. (2003). Fundamentals of Human Neuropsychology, 5th Edn New York: Worth

[B25] Ktori M. Pitchford N. J. (2009). Development of letter position processing: effects of age and orthographic transparency. J. Res. Read. 32, 180–198 10.1111/j.1467-9817.2009.01394.x

[B26] Laird A. R. McMillan K. M. Lancaster J. L. Kochunov P. Turkeltaub P. E. Pardo J. V. (2005). A comparison of label-based review and ALE meta-analysis in the Stroop task. Hum. Brain Mapp. 25, 6–21 10.1002/hbm.20136 15846823 PMC6871676

[B27] Leon-Carrion J. Garcia-Orza J. Perez-Santamaria F. J. (2004). Development of the inhibitory component of the executive functions in children and adolescents. Int. J. Neurosci. 114, 1291–1311 10.1080/00207450490476066 15370187

[B28] Luna B. (2009). Developmental changes in cognitive control through adolescence. Adv. Child Dev. Behav. 37, 233–278 10.1016/S0065-2407(09)03706-9 19673164 PMC2782527

[B29] Luna B. Sweeney J. A. (2004). The emergence of collaborative brain function: FMRI studies of the development of response inhibition. Ann. N. Y. Acad. Sci. 1021, 296–309 10.1196/annals.1308.035 15251900

[B30] MacLeod C. M. (1991). Half a century of research on the Stroop effect: an integrative review. Psychol. Bull. 109, 163–203 10.1037/0033-2909.109.2.163 2034749

[B31] MacLeod C. M. (2005). “The Stroop task in cognitive research,” in Cognitive Methods and Their Application to Clinical Research, eds Wenzel A. Rubin D. C. (Washington: American Psychological Association), 17–40

[B32] Marmurek H. H. Proctor C. Javor A. (2006). Stroop-like serial position effects in color naming of words and nonwords. J. Exp. Psychol. 53, 105–110 10.1027/1618-3169.53.2.105 16909934

[B33] Marsh R. Zhu H. Schultz R. T. Quackenbush G. Royal J. Skudlarski P. (2006). A developmental fMRI study of self-regulatory control. Hum. Brain Mapp. 27, 848–863 10.1002/hbm.20225 16421886 PMC2292452

[B34] McCown D. A. Arnoult M. D. (1981). Interference produced by modified Stroop stimuli. Bull. Psychon. Soc. 17, 5–7

[B35] Montgomery D. E. Koeltzow T. E. (2010). A review of the day-night task: the Stroop paradigm and interference control in young children. Dev. Rev. 30, 308–330 10.1016/j.dr.2010.07.001

[B36] Paris S. (2005). Reinterpreting the development of reading skills. Read. Res. Q. 40, 184–202 10.1598/RRQ.40.2.3

[B37] Perea M. Estevez A. (2008). Transposed-letter similarity effects in naming pseudowords: Evidence from children and adults. Eur. J. Cogn. Psychol. 20, 33–46 10.1080/09541440701306941

[B38] Peressotti F. Mulatti C. Job R. (2010). The development of lexical representations: evidence from the position of the diverging letter effect. J. Exp. Child Psychol. 106, 177–183 10.1016/j.jecp.2010.02.002 20211474

[B39] Peru A. Faccioli C. Tassinari G. (2006). Stroop effects from 3 to 10 years: the critical role of reading acquisition. Arch. Ital. Biol. 144, 45–62 16425617

[B40] Peterson B. S. Potenza M. N. Wang Z. Zhu H. Martin A. Marsh R. (2009). An FMRI study of the effects of psychostimulants on default-mode processing during Stroop task performance in youths with ADHD. Am. J. Psychiatry 166, 1286–1294 10.1176/appi.ajp.2009.08050724 19755575 PMC3289412

[B41] Pocklington B. Maybery M. (2006). Proportional slowing or disinhibition in ADHD? A Brinley plot meta-analysis of Stroop color and word test performance. Int. J. Disabil. Dev. Educ. 53, 67–91 10.1080/10349120500510057

[B42] Polderman T. J. de Geus E. J. Hoekstra R. A. Bartels M. van Leeuwen M. Verhulst F. C. (2009). Attention problems, inhibitory control, and intelligence index overlapping genetic factors: a study in 9-, 12-, and 18-year-old twins. Neuropsychology 23, 381–391 10.1037/a0014915 19413451

[B43] Pritchard V. E. Neumann E. (2004). Negative priming effects in children engaged in nonspatial tasks: evidence for early development of an intact inhibitory mechanism. Dev. Psychol. 40, 191–203 10.1037/0012-1649.40.2.191 14979760

[B44] Redding G. M. Gerjets D. A. (1977). Stroop effect: interference and facilitation with verbal and manual responses. Percept. Mot. Skills 45, 11–17 10.2466/pms.1977.45.1.11 905071

[B45] Reeve W. V. Schandler S. L. (2001). Frontal lobe functioning in adolescents with attention deficit hyperactivity disorder. Adolescence 36, 749–765 11928880

[B46] Regan J. (1978). Involuntary automatic processing in color-naming tasks. Atten. Percept. Psychophys. 24, 130–136 10.3758/BF03199539 693247

[B47] Schachar R. Logan G. D. (1990). Impulsivity and inhibitory control in normal development and childhood psychopathology. Dev. Psychol. 26, 710–720 10.1037/0012-1649.26.5.710

[B48] Schiller P. H. (1966). Developmental study of color-word interference. J. Exp. Psychol. 72, 105–108 10.1037/h0023358 5967713

[B49] Schwartz K. Verhaeghen P. (2008). ADHD and Stroop interference from age 9 to age 41 years: a meta-analysis of developmental effects. Psychol. Med. 38, 1607–1616 10.1017/S003329170700267X 18226285

[B50] Singer M. H. Lappin J. S. Moore L. P. (1975). The interference of various word parts on color naming in the Stroop test. Percept. Psychophys. 18, 191–193 10.3758/BF03205966

[B51] Starrfelt R. Gerlach C. (2007). The visual what for area: words and pictures in the left fusiform gyrus. Neuroimage 35, 334–342 10.1016/j.neuroimage.2006.12.003 17239621

[B52] Stirling N. (1979). Strrop Interference: An input and an output phenomenon. Q. J. Exp. Psychol. (Hove) 31, 121–132

[B53] Stroop J. R. (1935). Studies of interference in serial verbal reactions. J. Exp. Psychol. 18, 643–662 10.1037/h0054651

[B54] van Mourik R. Oosterlaan J. Sergeant J. A. (2005). The Stroop revisited: a meta-analysis of interference control in AD/HD. J. Child Psychol. Psychiatry 46, 150–165 10.1111/j.1469-7610.2004.00345.x 15679524

[B55] Vellutino F. R. Tunmer W. E. Jaccards J. J. Chen R. (2007). Components of reading ability: Multivariate evidence for a convergent skills model of reading development. Sci. Stud. Read. 11, 3–32 10.1080/10888430709336632

[B56] White B. W. (1969). Interference in identifying attributes and attribute names. Percept. Psychophys. 6, 166–168 10.3758/BF03210086

[B57] White D. A. Nortz M. J. Mandernach T. Huntington K. Steiner R. D. (2001). Deficits in memory strategy use related to prefrontal dysfunction during early development: evidence from children with phenylketonuria. Neuropsychology 15, 221–229 10.1037/0894-4105.15.2.221 11324865

[B58] Williams B. R. Ponesse J. S. Schachar R. Logan G. D. Tannock R. (1999). Development of inhibitory control across the lifespan. Dev. Psychol. 35, 205–213 10.1037/0012-1649.35.1.205 9923475

[B59] Wright B. C. Wanley A. (2003). Adults’ versus children’s performance on the Stroop task: interference and facilitation. Br. J. Psychol. 94(Pt 4), 475–485 10.1348/000712603322503042 14687456

